# Salmonella serovar Infantis REPJFX01 isolates bear a pESI plasmid that includes additional genes not found in closely related non-REP strains

**DOI:** 10.1099/mgen.0.001523

**Published:** 2025-10-07

**Authors:** Anna Schumann, Martin Wiedmann, Renato H. Orsi

**Affiliations:** 1 Department of Food Science, Cornell University, Ithaca, New York, USA; 2 Graduate Field of Biomedical and Biological Sciences, Cornell University, Ithaca, New York, USA

**Keywords:** antimicrobial resistance, plasmid of emerging *S*. Infantis (pESI) plasmid, reoccurring, emerging and persistent (REP) strains, *Salmonella* Infantis

## Abstract

*Salmonella enterica* subsp. *enterica* serovar Infantis (*S*. Infantis) REPJFX01 is a multidrug-resistant strain associated with poultry and international travel; this strain has been designated as a reoccurring, emerging and persistent (REP) strain by the US Centers for Disease Control and Prevention due to its identification in a number of foodborne illnesses and outbreaks. We compared publicly available *S*. Infantis REPJFX01 genomes to genomes of non-US isolates from the same NCBI SNP cluster and non-REP *S*. Infantis isolates from four other SNP clusters. When compared to isolates from the non-REP group, 135 genes were identified to be significantly (*P*<0.001) associated with REPJFX01; 50 of these genes were located on plasmid of emerging *S*. Infantis. Of the 135 REP-associated genes, 29 genes were functionally annotated and included transcription, recombination, arsenic and antimicrobial resistance genes. This suggests that the acquisition of mobile elements containing resistance genes may have favoured the emergence and spread of REPJFX01. Our results show that REPJFX01 is distributed in both North and South America, indicating that international perspectives on REP strains may be valuable.

**Impact Statement**

*Salmonella enterica* subsp. *enterica* serovar Infantis (*S*. Infantis) REPJFX01 was designated as a reoccurring, emerging and persistent (REP) strain by the US Centers for Disease Control and Prevention to better monitor its spread and changes over time. To determine how *S*. Infantis REPJFX01 differs from other *S*. Infantis strains, we performed genome-wide association studies and showed that (i) certain plasmid of emerging *S*. Infantis (pESI) located genes, but not the pESI plasmid itself, are associated with REPJFX01 strains and that (ii) US REPJFX01 isolates and closely related isolates from South America likely represent a single REP strain. Our findings indicate that REPJFX01 is internationally distributed and suggest that international efforts might be needed to reduce the spread of REPJFX01. Additionally, the presence of several resistance genes in the vicinity of transposons highlights the need to lower the spread of REPJFX01 to prevent spreading resistance genes to other bacteria.

## Data Summary

All accession numbers for genomes and all protocols used in these studies are provided within the article or supplementary tables.

## Introduction

Non-typhoidal *Salmonella* spp. cause the second largest number of bacterial foodborne illnesses in the USA [1,2]. *Salmonella enterica* subsp. *enterica* serovar Infantis (*S*. Infantis) is oftentimes linked to poultry, specifically chicken [3]. Importantly, the prevalence of *S*. Infantis isolated from chicken meat has been increasing in the past decade in the USA [4]; the prevalence of *S*. Infantis among *Salmonella* isolates in retail chicken meat increased from 2.8 to 39.7% from 2015 to 2020 [5]. Additionally, *S*. Infantis causes high numbers of salmonellosis in humans in Europe and the USA [6]. Based on FoodNet Fast [7], *S*. Infantis accounted for 2.9% of infections in culture-confirmed *Salmonella* isolates in 2022, representing the seventh most common serovar among human clinical cases, compared to 1.7% in 2011 when it represented the ninth most common serovar among human clinical cases. In the past few years, *S*. Infantis has also emerged as a multidrug-resistant (MDR) pathogen of concern not only in the USA but also in the Americas, Europe and Asia [6,8, 9].

Among MDR *S*. Infantis isolates, those carrying the megaplasmid pESI (‘plasmid of emerging *S*. Infantis’) have been of particular concern, given that this plasmid frequently carries mobile genetic elements and antimicrobial resistance (AMR) genes [6,10]. Several studies have reported the presence of a more recent group of plasmids referred to as pESI-like plasmids in *S*. Infantis, carrying several AMR genes, including the extended-spectrum *β*-lactamase (ESBL) gene *bla_CTX-M-65_* [6,10]. This is concerning given that people with severe *Salmonella* infections or with a high risk of developing invasive infections are treated with cephalosporins or penicillins, to which CTX-M-65 confers resistance [11,12]. Because there are no clear definitions of pESI and pESI-like plasmids, the term ‘pESI’ will be used here to refer to both pESI and pESI-like plasmids unless otherwise noted.

To better understand which enteric bacterial strains contribute to severe illnesses and outbreaks within the USA, the Centers for Disease Control and Prevention (CDC) has introduced reoccurring, emerging and persistent (REP) strain designations [13]; the overall goal is to find new measures to reduce the prevalence of REP strains and foodborne illnesses in general. *S*. Infantis REPJFX01 has been designated as a REP strain due to its persistence within the USA since 2012 [14]. The spread of REPJFX01 to humans has been attributed to consumption of contaminated chicken and international travel, specifically to the Dominican Republic or Peru [14]. Additionally, between 2018 and 2022, 73% of the REPJFX01 human clinical isolates harboured AMR genes that rendered the recommended antibiotic treatment ineffective [14].

Because of the importance of *S*. Infantis REPJFX01 to public health, we sought to determine whether the presence of certain genes is associated with *S*. Infantis REPJFX01 in comparison to closely related *S*. Infantis isolates. Improving our understanding of genetic characteristics specific to *S*. Infantis REPJFX01 might help with the identification of new intervention and/or control strategies that can reduce REPJFX01’s spread.

## Methods

### Selection of US REP, SA REP and non-REP strain genomes and annotation of assemblies

Single nucleotide polymorphism (SNP) clusters for genome-wide association studies (GWAS) were selected based on *S*. Infantis phylogeny described by Chen *et al*. [15]. We defined three different groups for GWAS: (i) a ‘US REP’ group that contained only isolates from SNP cluster PDS000184420 and isolation locations within the USA, and two sister groups for comparison: (ii) a ‘South America (SA) REP’ group that contained isolates from SNP cluster PDS000184420 with isolation locations from Peru, Ecuador and Chile and (iii) a ‘non-REP’ group that contained an equal number of isolates from SNP clusters PDS000174351, PDS00032463, PDS0003948 and PDS85033 (from clades that clustered most closely with the US/SA REP strain clade in the phylogeny described by Chen *et al*. [15]) with listed isolation locations. For all groups, only metadata from isolates fulfilling the following assembly quality criteria were downloaded from National Center for Biotechnology Information (NCBI) Pathogen Detection (30/07/2024): (i) contig number: 2–100, (ii) N50: >100,000 bp and (iii) genome length: >4.5 Mbp, <5.5 Mbp.

A random number generator was used to select 300 isolates for GWAS by selecting 100 isolates from each of (i) the US REP and (ii) SA REP groups and 25 isolates from each of the four SNP clusters making up the non-REP group (referred to as the non-REP group). All genome assemblies were downloaded from NCBI Pathogen Detection (02/08/2024) and annotated using Prokka v.1.14.5 [16]. Group associations and metadata of all analyzed strains can be found in Table S1.

### Identification of genes associated with US REP strains

**Fig. 1.**
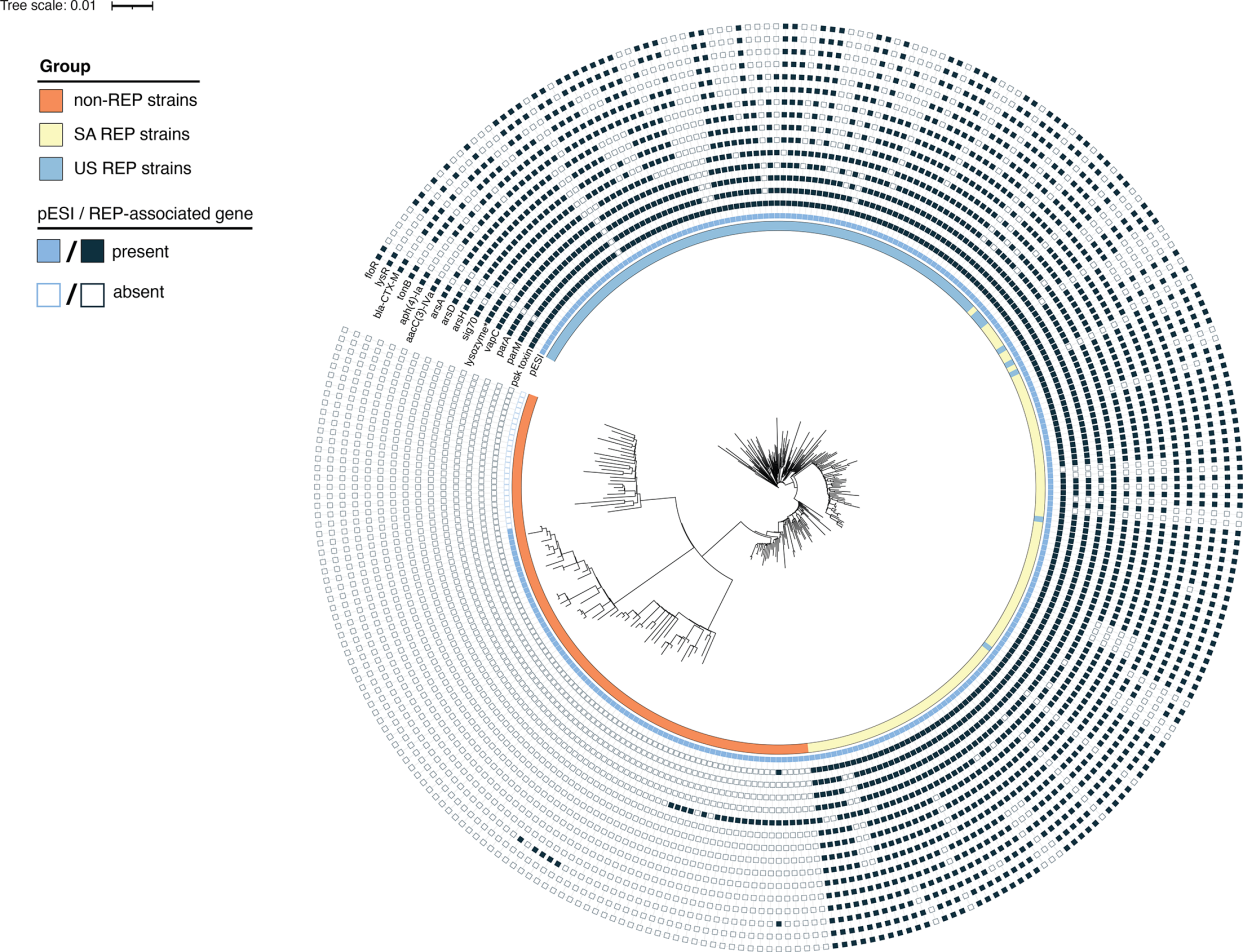
Maximum likelihood phylogeny of analysed *S*. Infantis isolates indicating the presence or absence of REP-associated genes. The maximum likelihood phylogeny of analysed *S*. Infantis isolates was constructed as described in Fig. 2. Group designations are shown on the innermost ring. Each ring shows whether (**i**) the pESI plasmid or (ii) chosen REP-associated genes are present or absent in each isolate. For *bla_CTX-M_* genes, variant classifications are *bla_CTX-M-65_* for US REP and SA REP isolates, *bla_CTX-M-1_* for non-REP isolates from the PDS00032463 SNP cluster and *bla_CTX-M-15_* for non-REP isolates from the PDS000174351 SNP cluster. Several genes (14/29, e.g. transcriptional regulators, transposons and integrases) are not shown but can be found in Tables 1 and S2.

To assess genome-wide associations based on the groups of isolates (US REP, SA REP and non-REP), Panaroo v.1.5.0 [17] and Scoary v.1.6.16 [18] were used. Genes were considered to be associated with US REP strains when *P*<0.05 (Bonferroni corrected) and the proportion difference ≥0.35 (determined by subtracting the proportion of non-REP isolates with a gene from the proportion of REP isolates with a gene). Scary outputs can be found in Tables S3 and S4 (available in the online Supplementary Material); for the US REP vs. non-REP analysis, ten plasmid-located genes were removed from the list, as the genes appeared twice with identical sequences, likely due to issues with either contig assembly or annotation. All US REP-associated functionally annotated genes were further analysed using the basic local alignment search tool (blast) [19] by comparing their translated nucleotide sequences against the NCBI non-redundant protein database to confirm annotations generated by Prokka [16]. Prokka-generated annotations were changed to annotations of blast hits if a REP-associated gene matched >10 blast hits with similar annotations (>99% query coverage, >99% identity). Changes in annotations from original Prokka [16] annotated genes to annotations used in Table 1 and Fig. 1 can be found in Table S5. For *bla_CTX-M_* variant assignment across our comparison groups, the ‘AMR genotypes core’ classification from NCBI Pathogen Detection metadata was used.

### Assessment of pESI presence in chosen assemblies

To determine whether isolates carried the pESI megaplasmid, genome assemblies were screened based on McMillan *et al*.’s [10] approach with a minor modification. Genome data of isolates were screened for 13 target sequences common to the pESI plasmid [*ardA*, I1 relaxase, *sogS*, *trbA*, pESI *repA*, IncP, pESI hypothetical backbone, K88, *ybt*, *merA* (two sequences), *ipF*, *pilL*] using blast [19]; the *bla_CTX-M-65_* genetic sequence was not included in our screen. Briefly, isolates were considered to carry a pESI plasmid if they contained the pESI *repA* gene and five additional target sequences with >95% coverage and >95% identity in comparison to the target sequence.

### Phylogenetic tree construction and sequence type determination

kSNP v. 4.0 [20] was used to identify pan-genome SNPs among all of our 300 isolates within the three groups using a *k*-mer size of 19 nt; those SNPs were subsequently used to construct a phylogenetic tree using kSNP v. 4.0 [20]. Core SNPs determined by kSNP v. 4.0 [20] were also used to determine the number of SNP differences between isolates within each comparison group and between different comparison groups. The phylogenetic tree was visualized and edited using the Interactive Tree of Life v.7 [21]. Sequence types of isolates were determined using the programme mlst v.2.23.0 [22] with the ‘senterica_achtman_2’ scheme. Downloaded metadata from NCBI Pathogen Detection were used to determine isolation locations, isolation sources and the number of AMR gene classes (data used for the tree annotation can be found in Table S2).

### Identification of REP strain-associated gene location

To determine the loci of US REP-associated functionally annotated genes, the assembly of isolate FSIS21720811 (accession: GCA_009594345; three contigs, N50=3,895,539 bp) was used, as it contained 29/29 and 99/106 of the US REP annotated and hypothetical genes, respectively, and only consisted of three contigs: two for the chromosome and one for the plasmid. Each US REP-associated gene was assigned a chromosomal or plasmid location based on the Panaroo output for FSIS21720811, and all US REP-associated functionally annotated genes were assigned loci based on the FSIS21720811 genome assembly using the software Geneious v. 2024.0.5.

## Results

### *S*. Infantis isolates representing US REP and SA REP groups are more closely related to each other than to isolates from the non-REP group

**Fig. 2.**
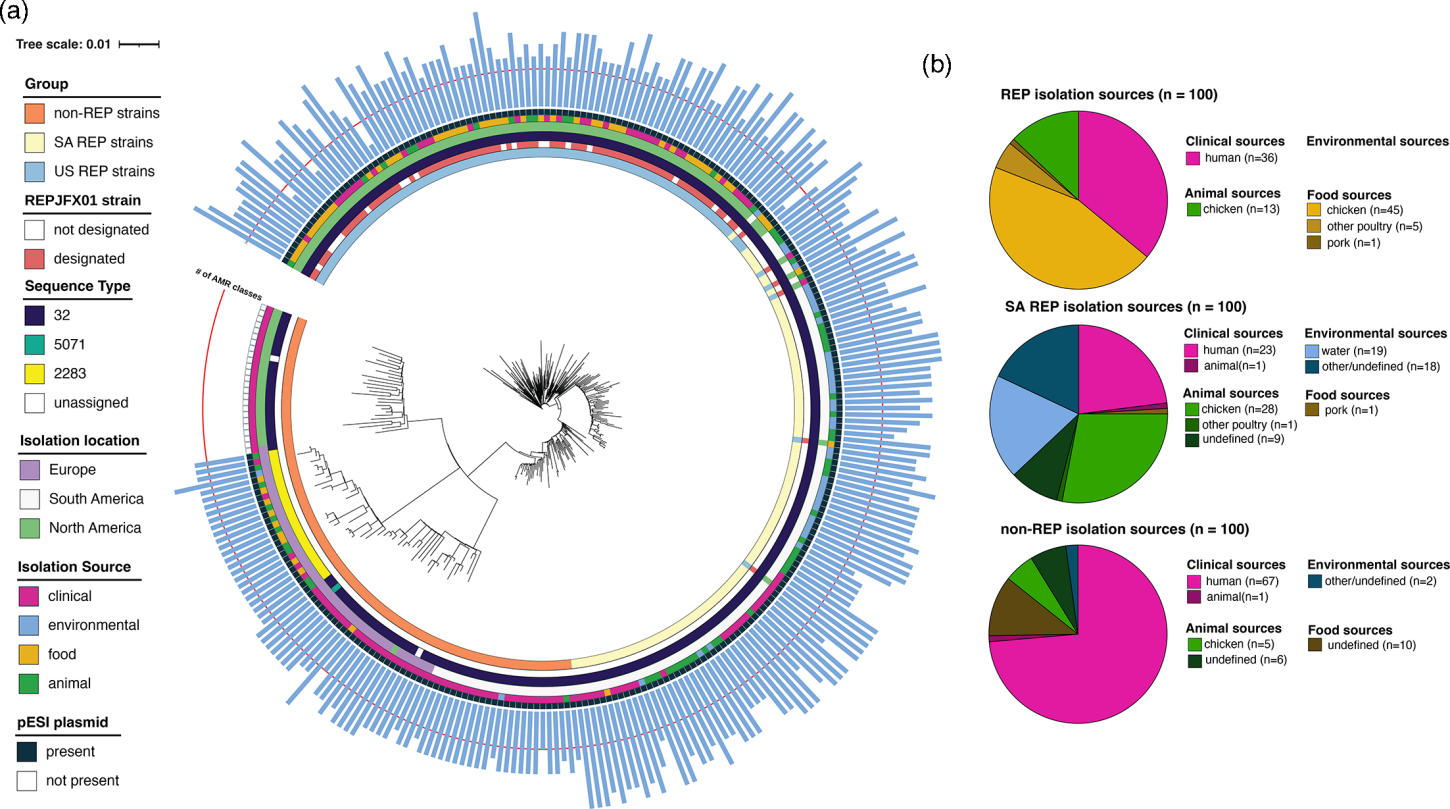
Maximum likelihood phylogeny of analysed *S*. Infantis isolates and their isolation data. (**a**) The maximum likelihood phylogeny of analysed *S*. Infantis isolates was constructed based on core SNPs identified among all 300 isolates; branch lengths represent the average pairwise nucleotide substitutions per site. Group designations, designation as REPJFX01 (based on personal communication with the US CDC), sequence types (ST 5071 and ST 2283 differ from ST 32 by one allele), isolation location, isolation source and presence of the pESI plasmid are indicated. The outermost ring shows the number of AMR classes present in each isolate, with the red line being set to *n*=3. Isolates whose outermost bar touches or exceeds the red line are considered MDR. (**b**) Identification of sub-classes for clinical, food and animal isolates across all three groups (US REP, SA REP and non-REP). Environmental isolates (*n*=39) include 19 isolates from water sources but are not shown in the figure.

The three comparison groups of *S*. Infantis isolates (i.e. US REP, SA REP and non-REP groups) were found to be closely related, as evident by the majority of the isolates representing ST 32 (*n*=272); other isolates represented ST 5071 (*n*=1), ST 2283 (*n*=25) or could not be assigned to an ST (*n*=2); however, ST 5071 and ST 2283 only differ from ST 32 by one allele, and all three STs are classified into Clonal Complex 31. When comparing our 300 isolates to 12,577 isolates classified as REPJFX01 by the CDC (CDC, pers. Comm., email, 28 March 2025), 80 of the 100 US REP isolates, but none of the SA REP or non-REP isolates, were recognized as REPJFX01 isolates (Fig. 2a). Additionally, all 100 analysed isolates from the US REP group only differed by 6–95 core SNPs. Isolates within the SA REP and non-REP groups were also closely related and differed by 0–72 and 0–171 core SNPs within each group, respectively. Isolates in the US and SA REP groups are more closely related to each other than to the isolates in the non-REP group, as some US REP isolates clustered with SA REP isolates, while all non-REP isolates clustered separately in the phylogenetic tree (Fig. 2a). Additionally, US REP isolates differed from SA REP and non-REP isolates by 4–97 (median=52) and 18–211 (median=136) core SNPs, respectively.

While all US and SA REP isolates were isolated from the USA and South America, respectively, non-REP isolates were obtained from Europe, North America and South America (Fig. 2a). Each of the three groups included isolates from clinical, food and animal sources; only the SA REP and non-REP groups included isolates from environmental sources (e.g. water, raw pet food and boot swabs; Fig. 2a, b). Most of the 100 US REP group isolates were of clinical (*n*=36) and food (*n*=51) origin; the 100 SA REP group isolates represented clinical (*n*=24), environmental (*n*=37; including 19 from water sources) and animal (*n*=38) sources, including 28 isolates from chickens. The 100 non-REP group isolates represented clinical (*n*=77), food (*n*=10) and animal (*n*=13) origins.

All US and SA REP isolates were found to carry the pESI plasmid; 75 of the 100 non-REP isolates also carried the pESI plasmid (Fig. 2a, Table 1). In our pESI analysis, US REP isolate REP_GCA_032587535 was found to not fulfil our screening criteria for pESI plasmids (i.e. detection of *repA* and 5 out of 13 target genes with specific coverage and identity) due to lack of coverage of three of the five identified target genes (likely due to the assembly having 97 contigs). Because we identified four other REP-associated, pESI-located genes in further analyses, we considered it to contain the pESI plasmid. Out of 300 analysed *S*. Infantis isolates, 275 isolates carried at least one AMR gene, and 268 isolates were classified as MDR (Fig. 2a). Only 25 non-REP isolates, all from SNP cluster PDS000085033, carried no AMR genes; these are the same isolates that did not carry a pESI plasmid (Fig. 2a).

**Table 1.** Prevalence of REP-associated genes and genetic features in all analysed strains by groups (genes are sorted based on prevalence in US REP strains)*

REP-associated gene	More detailed information	Location	US REP (*n*=100)	SA REP (*n*=100)	non-REP (*n*=100)§				*P* value US REP vs. non-REP comparison‖
					PDS0003948 (*n*=25)	PDS00032463 (*n*=25)	PDS000174351 (*n*=25)	PDS000085033 (*n*=25)	
pESI	Plasmid	na	100	100	25	25	25	0	na
IS3 family transposase†	–	P	100	94	23	24	18	0	3.02E-09
psk toxin	Post-segregational killing protein, Hok/Gef family protein	P	99	99	0	0	1	0	4.28E-52
Tyrosine recombinase/integrase†	–	P	98	100	0	0	0	0	2.21E-52
Tyrosine recombinase/integrase†	–	C	97	100	0	0	20	0	6.73E-29
Transposase†	–	P	97	91	20	13	15	10	1.34E-08
*parM*	Plasmid segregation protein ParM	P	94	94	0	0	0	0	7.31E-47
*parA*	AAA family ATPase, plasmid segregation protein ParA	P	93	94	0	0	0	0	1.12E-45
Integrase†	–	P	93	94	0	0	0	0	1.12E-45
Transcriptional regulator†	–	C	88	100	0	0	20	0	4.72E-20
Transcriptional regulator†	–	P	87	93	0	0	0	0	1.64E-39
*vapC*	tRNA(fMet)-specific endonuclease VapC	P	84	89	0	0	0	0	7.44E-37
Helicase†	Replicative DNA helicase	C	82	88	0	9	19	5	4.09E-09
Lysozyme	Lysozyme/phage endolysin	C	80	90	0	0	20	0	1.31E-14
IS630 family transposase†	–	P	80	78	11	9	8	0	2.40E-10
*sig70*-like sigma factor	Sigma-70 RNA polymerase sigma factor	P	75	84	0	0	0	0	5.57E-30
*arsH*	NADPH-dependent FMN reductase ArsH	P	75	84	0	0	0	0	5.57E-30
*arsD*	Arsenical resistance operon *trans*-acting repressor ArsD	P	73	84	0	0	0	0	1.27E-28
*arsA*	Arsenical pump-driving ATPase	P	72	84	0	0	0	0	5.8E-28
*aacC(3)-IVa*	Aminoglycoside *N*-acetyltransferase AAC(3)-IVa (aminoglycoside resistance)	P	68	85	0	0	0	0	1.95E-25
*aph(4)-Ia*	Aminoglycoside *O*-phosphotransferase (APH(4)-Ia) (hygromycin resistance)	P	67	85	0	0	0	0	7.86E-25
IS903B family transposase†	–	P	64	78	0	0	0	0	4.51E-23
IS6 family transposase†	–	P	64	85	0	0	0	0	4.51E-23
Tn3 family transposase†	–	P	63	85	0	0	0	0	1.67E-22
*tonB*	*tonB*-dependent receptor	P	54	77	0	0	0	0	9.8E-18
*bla_CTX-M_*‡	Beta-lactamase CTX-M‡	P	54	77	0	5	1	0	5.09E-11
*lysR*	LysR family transcriptional regulator	P	53	82	0	0	0	0	3.06E-17
*floR*	Chloramphenicol/florfenicol efflux MFS transporter floR	P	53	82	0	0	0	0	3.06E-17
*istB*†	ISEc57 family helper ATPase IstB	P	53	83	0	0	0	0	3.06E-17
IS91 family transposase†	–	P	48	46	0	0	0	0	7.46E-15

* –, Not available; na, not applicable; P, genetic feature located on the pESI plasmid; C, genetic feature located on chromosome.

† Genes not mapped onto the phylogenetic tree (Fig. 1).

‡ REP and SA REP strains carry *bla_CTX-M-65_*; non-REP strains from SNP cluster PDS00032463 and PDS000174351 carry *bla_CTX-M-1_* and *bla_CTX-M-15_*, respectively.

§ SNP cluster numbers are based on the phylogenetic tree from Chen *et al*.’s [15] study that were used to choose isolates for analysis and may differ from current SNP cluster assignments.

‖Adjusted *P* values based on Bonferroni’s correction.

### Genes involved in recombination, gene modulation, arsenic and AMR, but not pESI carriage, are associated with *S*. Infantis US REP isolates

When comparing genomes from isolates within the US and SA REP groups, we did not find any genes that were associated with either US or SA REP isolates (i.e. with proportion difference of 0.35 or larger; Table S4). However, 135 genes were found to be significantly associated with the US REP strain isolates, as compared to non-REP isolates (Tables 1 and S3). While 16 genes were associated with non-REP isolates as compared to US REP isolates, those were not further analysed because we wanted to focus on genes associated with US REP isolates. Of the 135 genes found to be US REP-associated, 106 were genes annotated as encoding hypothetical proteins, and 29 genes had functional annotations (hereon referred to as ‘US REP-associated functionally annotated genes’). Sixteen of these genes are involved in genetic recombination and modulation and include genes annotated as encoding transposases (*n*=7), integrases (*n*=3), transcriptional regulators (*n*=3), a helicase (*n*=1) and a *sig70*-like sigma factor (*n*=1). The other US REP-associated functionally annotated genes included genes annotated as arsenic resistance genes (*n*=3), AMR genes (*n*=4; i.e. *aacC(3)-IVa*, *aph (4)-Ia*, *bla_CTX-M-65_* and *floR*), partitioning protein genes (*n*=2; i.e. *parA* and *parM*), toxins of toxin-antitoxin module genes (*n*=2; i.e. psk toxin and *vapC*), a *tonB*-dependent receptor gene (*n*=1) and a lysozyme gene (*n*=1) (Table 1, Fig. 1).

We also determined how many isolates within the SA REP and non-REP groups carried US REP-associated functionally annotated genes identified in the US REP vs. non-REP comparison (Table 1). All 29 US REP-associated functionally annotated genes were also found in isolates within the SA REP group. Frequently, the number of isolates carrying US REP-associated functionally annotated genes was similar for the US and SA REP groups; for example, 93/100 analysed US REP and 94/100 analysed SA REP isolates carried *parA* (Table 1). In contrast, 21 of the 29 US REP-associated functionally annotated genes were not found in any of the non-REP isolates. Genes encoding one of the integrases, two transposases, a transcriptional regulator, the lysozyme and a helicase were found in a small fraction of the non-REP isolates (Table 1). Interestingly, one non-REP isolate was found to carry the psk toxin (Table 1), and six of the non-REP isolates were found to carry an ESBL *bla_CTX-M_* variant. While *bla_CTX-M-65_* is frequently associated with *S*. Infantis MDR isolates and found in both US REP (*n*=54) and SA REP (*n*=77) isolates, five isolates from SNP cluster PDS00032463 and a single isolate from SNP cluster PDS000174351 carried *bla_CTX-M-1_* and *bla_CTX-M-15_*, respectively.

### Most US REP-associated functionally annotated genes are located around three plasmid loci

**Fig. 3.**
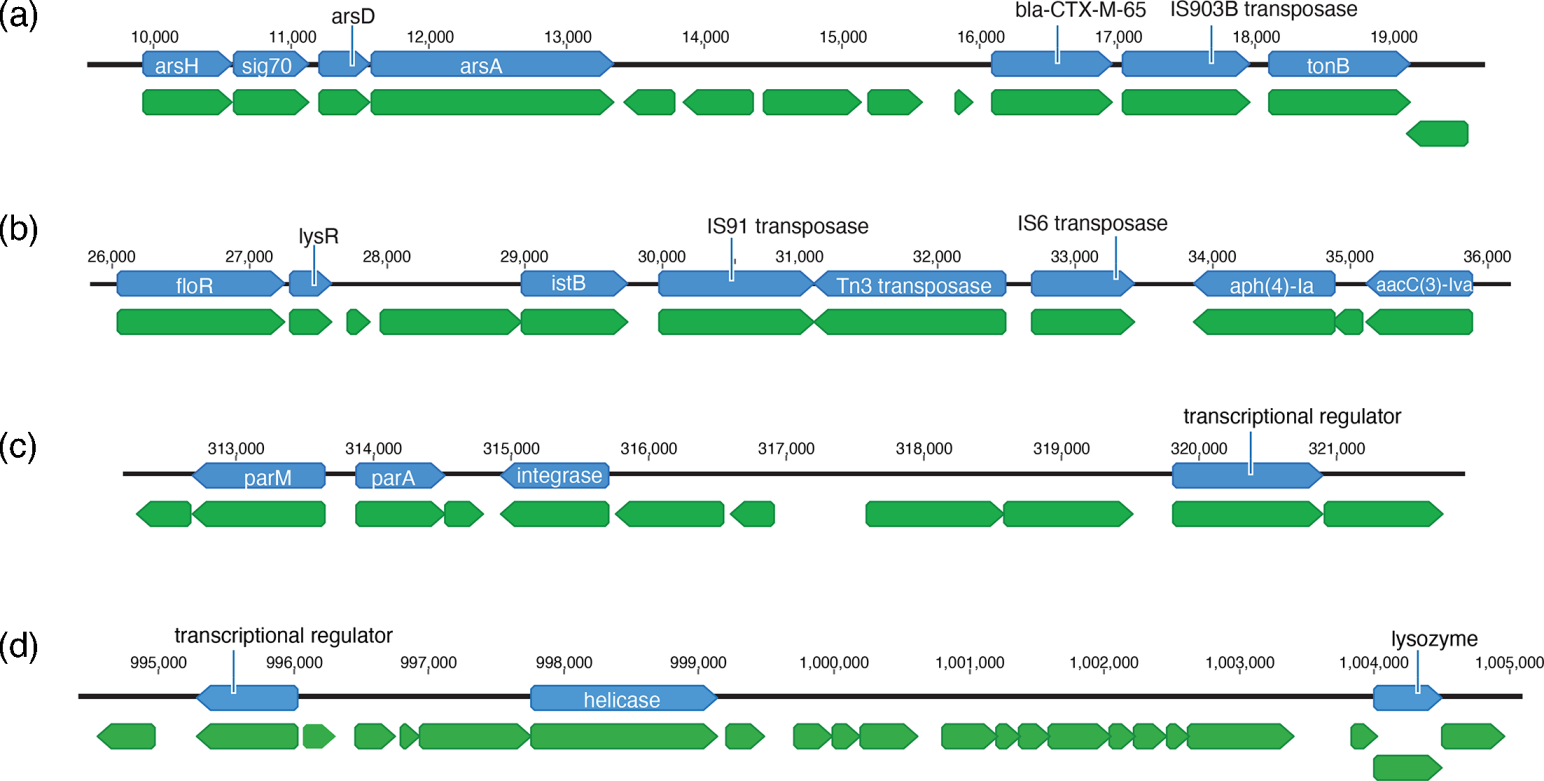
REP-associated genes and their likely location on the pESI plasmid and the chromosome. Loci containing REP-associated genes on the (**a–c**) pESI plasmid and the (d) *S*. Infantis chromosome based on their location on a closed REP isolate (accession number: GCA_032587535), a US REP isolate with an almost completed genome and a completed pESI plasmid containing all REP-associated genes. All identified genes around each locus are shown in green; REP-associated genes are shown in blue with blast-based annotations.

Because plasmids allow for easy dissemination of AMR and virulence-associated genes, we assessed whether US REP-associated genes were located on pESI. We used the almost completed assembly for the US REP isolate FSIS21720811 (accession: GCA_009594345), which contained all US REP-associated functionally annotated genes and most hypothetical protein genes, to map each gene’s location (corresponding gene numbers in FSIS21720811 of REP-associated genes are listed in Table S3). Out of the 106 hypothetical genes, 25 are located on the plasmid, 74 are located on the chromosome and for 7 we were not able to determine the location (Table S3). The majority (25/29) of the identified US REP-associated functionally annotated genes are likely located on pESI; 19 of these plasmid-located genes fall within three distinct loci, referred to as A, B and C (Fig. 3). Two of the loci (A, B) include US REP-associated transposases (Fig. 3a, b), with none of those transposases found in any of the non-REP isolates (Table 1). The third locus, C, did not include any evidence of US REP-associated transposases or mobile elements (Fig. 3c). Additionally, most of the chromosomally located US REP-associated functionally annotated genes (3/4) are located in proximity to each other (Fig. 3d).

A closer examination of the three identified plasmid loci shows that genes with similar functions cluster together. For example, in locus A, three genes involved in arsenic resistance (i.e. *arsH*, *arsD* and *arsA*) are located closely to each other (Fig. 3a). In locus B, two AMR genes (*aph(4)-Ia* and *aacC(3)-IVa*) and several genes involved in transposition (i.e. *istB* and three REP-associated transposases) are located closely to each other (Fig. 3b). Lastly, in locus C, *parA*, *parM* and an integrase cluster together (Fig. 3c).

## Discussion

*S*. Infantis REPJFX01 has been designated as a persistent strain by the US CDC and has been reported to be associated with contaminated chicken and international travel [14]. By performing GWAS between US REP isolates and isolates within two comparison groups, i.e. SA REP and non-REP isolates, we found (i) that isolates in the US and SA REP groups are part of a single *S*. Infantis REP strain, (ii) that the REP strain is characterized by the carriage of pESI-located genes but not pESI itself and (iii) that the REP strain-associated functionally annotated genes are involved in transposition, transcription, arsenic and AMR.

Consistent with chicken as one of the primary reservoirs for *S*. Infantis [6], the majority of the studied, randomly selected *S*. Infantis isolates obtained from food or animal sources originated from poultry, more specifically chicken. The US and SA REP isolates were in the same NCBI SNP cluster and showed a close relationship based on their phylogenetic clustering and the observed number of SNP differences. This likely explains why we were not able to identify any genes that were associated with either the US or SA REP isolates when comparing these two groups. Therefore, our findings suggest that it may be reasonable to consider all isolates that cluster with the US and SA REP groups as a single *S*. Infantis REP strain, which is consistent with epidemiological data that suggest travel to South America might have played a role in the spread of REPJFX01 [12,14]. Using tip-dated evolutionary analysis, Chen *et al*. [15] observed a clade of *S*. Infantis containing MDR isolates obtained exclusively in the USA on or after 2015. Although these isolates were not identified as REPJFX01 in their study, the isolates are in the same NCBI SNP cluster as the isolates in the US REP and SA REP groups, suggesting that they do represent REPJFX01. Interestingly, Chen *et al*. [15] also observed that these MDR US isolates share a common ancestor that existed circa 1990 with isolates from Ecuador and Peru obtained on or before 2015, suggesting that REPJFX01 may have originated in South America at the end of the 20th century.

While *S*. Infantis has not previously been reported as being associated with contaminated water, 19 of 100 *S*. Infantis isolates from the SA REP group were isolated from water sources. These findings are consistent with a global, systematic review identifying the environment as one of several reservoirs for MDR *S*. Infantis [6] and a study that found several MDR *Salmonella* serovars in rivers and irrigation canals in Chile [23]. Because MDR *Salmonella* were primarily isolated from water sources in rural zones, the authors [23] suggested that run-off from animal farms could potentially contribute to the environmental spread of *Salmonella*. Consistent with previous suggestions that agricultural runoff may contribute to the spread of AMR [24], it is possible that the spread of *S*. Infantis strains carrying REP-associated genes to wastewater or the environment may contribute to the spread and persistence of these strains. This could provide opportunities for the transfer of additional AMR genes to the pESI plasmid, as well as the transfer of the pESI plasmid to other *Salmonella* strains. The presence of pESI in isolates within *S*. Senftenberg and *S*. Alachua clusters has been shown in a previous study [25]. The authors suggested that the pESI plasmid possibly was transferred from *S*. Infantis to *S*. Senftenberg isolates, followed by transfer from *S*. Senftenberg to *S*. Alachua isolates [25].

Based on our results, it seems that US REP isolates (and REPJFX01 as a whole) are associated with certain pESI-located genes, but not the pESI plasmid, indicating the carriage of the newer, ‘pESI-like’ plasmid and not the original pESI plasmid. The ‘pESI-like plasmid’ has been identified as a circulating, newer version of the pESI plasmid, likely formed through insertion of several gene cassettes, like the *bla_CTX-M-65_* gene, or mobile elements over time [6,26]. Our results showed the association of seven transposases with US REP strains, thus supporting the notion that two different types of pESI plasmids are circulating worldwide [6]. Additionally, 50/135 REP-associated genes were likely located on pESI, and many of the functionally annotated genes were located around three loci. This further emphasizes that pESI is mosaic and highly plastic, suggesting pESI likely contributes to the evolution of REPJFX01. While we did not further analyse the 106 hypothetical US REP-associated genes, they should be further explored in future studies, including their role in REP strain emergence and persistence.

While our data suggest that *S*. Infantis US REP isolates may have acquired additional AMR genes, especially given that US REP-associated AMR genes were found to be closely located to transposase genes on pESI, we also confirmed that other *S*. Infantis isolates closely related to the US and SA REP groups appear to often be MDR. Out of our 300 analysed isolates, 268 were MDR and only 25 isolates did not carry any AMR genes, which is corroborated by other reports classifying REPJFX01 and other *S*. Infantis strains as MDR [6,14]. We found *bla_CTX-M-65_*, *aph(4)-Ia* and *aacC(3)-Iva* and *floR* genes to be associated with US REP isolates, which provide resistance to extended-spectrum *β*-lactams, aminoglycosides and amphenicols, respectively. Here, most US REP-associated AMR genes were unique to US and SA REP isolates and plasmid-located, though six non-REP isolates were identified to harbour plasmid-located *bla_CTX-M_* variants other than *bla_CTX-M-65_*. Another study [10] that examined the content of pESI plasmids within *S*. Infantis isolates found that 61% of all plasmids contained *bla_CTX-M-65_* and that other resistance genes like *aph(4)-Ia* and *floR* were detected in 55–72% of plasmids. In our dataset, 46, 51 and 45% of all analysed isolates carried *bla_CTX-M-65_*, *aph(4)-Ia* and *floR*, respectively.

Arsenic resistance genes were not only associated with US REP strains, but several previous studies have reported the occurrence of heavy metal resistance genes, providing resistance to arsenic [27,28] or mercury [26], in *S*. Infantis strains. Additionally, studies have shown that heterologous expression of *arsH* is sufficient to increase *Escherichia coli*’s resistance to reduced forms of aromatic arsenic like poultry growth promoter roxarsone [29], suggesting that the *ars* operon detected in US REP isolates could be sufficient to provide resistance to arsenic. While arsenic-containing drugs have been widely used in poultry husbandry in the USA until 2013 [30], arsenic has also been used in various other industries, e.g. as wood preservatives [31] and in pigments [32]. Due to the abundance of arsenic compounds in various environments, it is thought that almost all living organisms have mechanisms for arsenic detoxification, explaining the high abundance of arsenic resistance operons found in both Gram-positive and Gram-negative bacteria [33]. As exposure to heavy metals appears to facilitate co-selection of plasmid-located AMR and metal resistance genes [34,35], exposure to arsenic could have facilitated the co-selection of *S*. Infantis strains carrying arsenic and AMR genes, especially considering that the *ars* and *bla_CTX-M-65_* genes were found in the same loci. However, it is unclear which source of arsenic exposure is responsible for the potential co-selection.

Our data indicate that isolates that cluster with the US and SA REP groups may be part of a single *S*. Infantis REP strain, suggesting an international perspective on REPJFX01 and potentially other REP strains could be highly valuable. Because chickens appear to be a primary reservoir for *S*. Infantis REPJFX01 both in the USA and in other countries, intervention and control strategies focusing on limiting the spread of *S*. Infantis REPJFX01 in chickens may be valuable. Further implementation of strategies that limit environmental dispersal of *Salmonella*, and in particular *S*. Infantis REPJFX01, may be valuable (e.g. by controlling farm run-off). Continued emphasis on judicious use of antibiotics and other compounds that may facilitate selection of the pESI plasmid containing US REP-associated genes may also be valuable, especially considering that pESI-carrying strains have been shown to have enhanced virulence, are more pathogenic in *in vivo* mouse models [8] and are shed in higher numbers in chicken infection studies [9]. Lastly, it may be valuable to track further evolution of pESI plasmids to monitor future evolution of the *S*. Infantis REPJFX01 strain and potential transmission to other *Salmonella* serovars.

## Supplementary material

10.1099/mgen.0.001523 Table S1.

## Abbreviations

AMR: antimicrobial resistance
blast: basic local alignment search tool
CDC: Centers for Disease Control and Prevention
ESBL: extended-spectrum *β*-lactamase
GWAS: genome-wide association studies
MDR: multidrug-resistant
NCBI: National Center for Biotechnology Information
pESI: plasmid of emerging *S*. Infantis
REP: reoccurring, emerging and persistent
*S*. Infantis: *Salmonella enterica* subsp. *enterica* serovar Infantis
SNP: single nucleotide polymorphism
